# Melatonin Effects on Hard Tissues: Bone and Tooth

**DOI:** 10.3390/ijms140510063

**Published:** 2013-05-10

**Authors:** Jie Liu, Fang Huang, Hong-Wen He

**Affiliations:** 1 Guanghua School of Stomatology, Hospital of Stomatology, Sun Yat-sen University, Guangzhou 510055, China; E-Mail: serena2007lj@163.com; 2 Guangdong Provincial Key Laboratory of Stomatology, Guangzhou 510080, China

**Keywords:** melatonin, bone remolding, osteoporosis, tooth development, osseointegration

## Abstract

Melatonin is an endogenous hormone rhythmically produced in the pineal gland under the control of the suprachiasmatic nucleus (SCN) and the light/dark cycle. This indole plays an important role in many physiological processes including circadian entrainment, blood pressure regulation, seasonal reproduction, ovarian physiology, immune function, *etc.* Recently, the investigation and applications of melatonin in the hard tissues bone and tooth have received great attention. Melatonin has been investigated relative to bone remolding, osteoporosis, osseointegration of dental implants and dentine formation. In the present review, we discuss the large body of published evidence and review data of melatonin effects on hard tissues, specifically, bone and tooth.

## 1. Introduction

Melatonin is an endogenous hormone rhythmically produced in the pineal gland under the control of the suprachiasmatic nucleus (SCN) and the light/dark cycle [1–3]. Beyond that, it is also produced by several other tissues, amongst them the retina, thymus, spleen, ovaries, testicles, intestine and bone marrow [4,5]. Extrapineal melatonin secreted by specific organs is used locally as an autocoid or paracoid and does not enter circulation [4]. Melatonin from pineal gland does not act upon any specific target organ; it reaches all tissues and, due to its amphiphilicity, it easily enters all subcellular compartments, such as mitochondria and the nucleus [5–7].

Melatonin plays an important role in many physiological processes including circadian entrainment, blood pressure regulation, seasonal reproduction, ovarian physiology, immune function, *etc*. [8]. The mechanism of melatonin action also is various. In some cases, melatonin actions are mediated by the binding of the indoleamine to membrane receptors (MT_1_, MT_2_, MT_3_) or nuclear receptors (ROR/RZR) [9]. Moreover, because of its lipophilic properties, melatonin passes through cell membranes to gain access to subcellular organelles [7]. It also probably binds to some cytosolic proteins like kinase C, calmodulin and calreticulin [10–12].

Recently, the investigation and applications of melatonin in the hard tissues, bone and tooth, have received great attention. Melatonin has been investigated relative to bone remolding [13], osteoporosis [14,15], osseointegration of dental implants [5,16], and dentine formation [17]. In the present review, we discuss the large body of published evidence and review data of melatonin effects on hard tissues, namely, bone and teeth.

## 2. Bone and Tooth

Bone and tooth are the hard tissues of mammals. They are very similar in many aspects, such as their structure, composition, marker proteins, and mineralization process, *etc.*

Structurally, bone is composed by compact bone, spongy bone and bone marrow. Bone marrow, inside the spongy bone, provides abundant blood for bone activities. Tooth is composed by enamel, dentine, cementum and dental pulp which supply the nutrition for the tooth.

The main component of bone substance, enamel, dentine and cementum are mineralized extracellular matrices consisting of organic and inorganic substances. In all of the components (except enamel), the organic component is comprised of 90% (by weight) of type I collagen fibers and 10% of non-collagenous proteins. The inorganic phase is constituted by small crystals of a mineral alkaline character, hydroxyapatite (HA) [Ca_10_(PO_4_)_6_(OH)_2_] [18].

Type I collagen is the most abundant and important matrix molecule in bone, dentine and cementum. It forms a three-dimensional network into which non-collagenous proteins and the nucleation of hydroxyapatite crystals are deposited [19].

Although the non-collagenous proteins are minor constituents of the matrix, they have various proteins (including of BSP, DSP, DPP, OPN, *etc.*) and play an important role in regulating the mineralization process. Most of them are the mineralization promoters, inhibitors or signaling molecules. For example, bone sialoprotein (BSP) and dentin sialoprotein (DSP) are involved in the initial formation of HA; DPP enhances the formation and growth of HA, while osteopontin (OPN) inhibits the formation and growth of HA [20,21].

Bone, dentine, and cementum have many similarities in general, but also some differences. Throughout life, bone tissue undergoes a continuous process of bone remolding, which consists of new bone formation and already-formed bone resorption, but dentine and cementum are not subject to remodeling; on the contrary, they must retain their stability over a long period of time [20], whereas the dentine matrix is continuously deposited throughout life. In this context, it is also interesting to study differences in dentinogenesis and osteogenesis. Previous studies have revealed that there were not only similarities of melatonin effects on bone formation and dentine formation, but also some differences, which will be summarized respectively in the following.

## 3. Melatonin and Bone

### 3.1. Melatonin and Bone Remolding

Bone is a dynamic tissue undergoing remodeling throughout life, and this remodeling requires a balance between deposition of new bone by osteoblasts and resorption of old bone by osteoclasts [13].

Bone modeling requires the interaction between multiple bone cells (osteoblasts/osteoclasts/osteocytes) to renew, maintain, or adjust bone strength and/or mineral homeostasis in response to changing environmental influences [14]. There are four distinct phases to this process: activation, resorption, reversal, and formation with resorption and formation taking place via osteoclasts and osteoblasts, respectively [14]. Initially, osteoclast precursors are attracted to a particular area of bone surface, then differentiation into osteoclast, which is responsible to bone resorption by acidification and proteolytic digestion [18]. In the reversal phase, bone resorption transitions into bone formation, and osteoblast precursors are recruited to proliferate and differentiate into osteoblasts that invade the resorption area and begin to form new bone by secreting osteoid, which is eventually mineralized [14].

Bone remolding processes are mediated by hormones, cytokines, growth factors and other molecules [22]. One of the hormones modulating bone formation and resorption is melatonin. It is hypothesized that melatonin, perhaps through three principle actions, modulates bone metabolism. Firstly, melatonin directly affects the actions of osteoblast and osteoclast. Numerous studies documented that melatonin increases pre-osteoblast/osteoblast/osteoblast-like cell proliferation, promotes the expression of type I collagen and bone marker proteins (e.g., alkaline phosphatase, osteopontin, bone sialoprotein and osteocalcin), and stimulates the formation of a mineralized matrix in these cells [23–27]. Besides, melatonin inhibits the differentiation of osteoclasts via decreases in the expression of RANK mRNA and increases in both the mRNA and protein levels of osteo-protegerin [28,29]. Secondly, melatonin indirectly regulates bone metabolism through the interaction with systemic hormones (e.g., PTH, calcitonin, and estrogen) or other moleculars. Ladizesky *et al.* [15] revealed that estradiol treatment could prolong the effect of melatonin to augment bone remodeling in ovariectomized rats; it indicates that appropriate circulating estradiol levels might be needed for melatonin effects on bone. Thirdly, osteoclasts generate high levels of superoxide anions during bone resorption that contribute to the degradative process. Melatonin is a significant free-radical scavenger and antioxidant. It can clear up the free radicals generated by osteoclast during the bone resorption process and protect bone cells from oxidative attacks [18,30,31].

### 3.2. Melatonin and Bone Repair

Bone fracture and bone defect are the common bone disease which originate from trauma, neoplasm invasiveness, surgery, or as a secondary effect from some bone diseases. The repair of bone fracture and bone defect is an important process to maintain the integrity and function of the bone.

Bone repair is a complex and continuous process. Biologically, it takes place in three stages: inflammatory, proliferative and remodeling phases [32]. During these stages, a set of complex biochemical events take place, including inflammatory cell infiltration, angiogenesis, cell proliferation and differentiation, collagen deposition, granulation tissue formation and mineral matrix deposition, *etc*. [32,33]. Previous studies have indicated that melatonin may play an important role in the bone-healing process due to its antioxidant properties, regulation of bone cells, and promotion of angiogenesis actions [33,34].

In the early stage of bone fracture/defect healing, the inflammatory phase is characterized by clot formation, ischemia and reperfusion injury and inflammatory cells (leukocytes, macrophage and mast cells) infiltration [35]. During this period, neutrophils produce free oxygen radicals that initiate a chain reaction leading to cell membrane damage via lipid peroxidation [35,36]. It has a negative effect on fracture/defect healing. The pineal hormone melatonin is a significant free radical scavenger and antioxidant at both physiological and pharmacological concentrations. Halici *et al.* [33] carried out biochemical and histopathologic observation of the outcomes of intraperitoneal applications of melatonin (30 mg/kg/day) for accelerating bone fracture healing in a rat model. The authors found that malondialdehyde (MDA) levels (indicator of free-radical concentration), superoxide dismutase (SOD) activity and myeloperoxidase ((MPO) plays a fundamental role in oxidant production) in the melatonin group decreased at the early stage of fracture healing compare to the control group, and SOD activity returned to the first-day value after 28 days in the melatonin group. These findings indicate that the administration of melatonin maybe beneficial in suppressing the effects of free oxygen radicals and regulating antioxidant enzyme (SOD) activity, thereby accelerating bone formation in the fracture-healing process [33].

In the proliferative phase, angiogenesis, osteoblast and fibroblast differentiation, collagen deposition and formation of granulation tissue take place in this phase. As mentioned before in this review, melatonin promotes the osteoblast proliferation and differentiation and enhances the type I collagen deposition [23,24]. Additionally, a recent study revealed that melatonin promotes angiogenesis during repair of bone defect in rabbit [32]. They observed the commencement of neovascularization and a significant increase in the number of vessels in the melatonin group in the first two weeks, which were also accompanied by an increase in the length of cortical formation. A similar outcome was also found by Soybir *et al.* [37] who reported an increase in the number of blood vessels resulting from melatonin applications to wounds in rats. Angiogenesis is an important physiological process in bone wound healing. Yamada *et al.* [38] suggest that angiogenesis precedes osteogenesis. The regeneration of new bone was dependent on blood vessels for the supply of mineral elements and the migration of angiogenic and osteogenic cells into secluded spaces.

That melatonin influences the remolding process during the remolding phase has been elaborated clearly elsewhere [23–31].

### 3.3. Melatonin and Osteoporosis

Osteoporosis was defined as “a systemic skeletal disease characterised by low bone mass and microarchitectural deterioration of bone tissue, with a consequent increase in bone fragility and susceptibility to fracture” by the World Health Organization [39]. It has been a major public health problem for healthy adults over the age of 55 years and with a major prevalence in women. About 50% of women will go on to develop an osteoporotic fracture, compared to 25% of men [40]. Without an intervention strategy, it is likely that the amount of people with osteoporosis will increase threefold over the next 25 years because of an increase in the aging population worldwide [41]. Recent therapies include targeting bone-resorbing osteoclasts by use of bisphosphonates, estrogen, selective estrogen receptor modulators (SERM) and calcitonin to prevent further bone breakdown, and stimulating bone-forming osteoblasts by anabolic drugs (e.g., teriparatide) to increase bone mass [42,43]. However, these therapies are limited because of their negative side effects or high costs [44,45]. In this sense, melatonin as a complementary therapy for the prevention and treatment of osteoporosis should be considered, because of its modulating bone metabolism function, lack of side effects, and economical advantages.

Some studies revealed the possible etiologic role of melatonin in osteoporosis. Nocturnal plasma melatonin levels decline with age. It has also been reported that melatonin secretion decreases sharply during menopause, which is associated with post-menopausal osteoporosis [46,47]. A correlation between decreased plasma melatonin levels and an increased incidence of bone deterioration as seen in post-menopausal women has been examined [48]. Furthermore, Ostrowska *et al.* [49] found that a pinealectomy in rats promotes the induction of bone metabolism biomarkers. In addition, Feskanich *et al.* [50] reported that twenty or more years of nightshift work significantly increased the risk of wrist and hip fractures in post-menopausal women. Nightshift work leads to disturbances of melatonin secretion as well as severe circadian rhythm disruption. These observations taken together suggest that melatonin may be involved in the pathogenesis of osteoporosis.

At present, few clinical trials have focused on the possible therapeutic value of melatonin in the prevention/treatment of osteoporosis. Most experimental studies were performed in ovariectomized rats, as a model for menopause. Uslu *et al.* [51] described that melatonin treatment increased trabecular thickness and the trabecular area of vertebra and femur and cortical thickness of femur, which decreased after ovariectomy in rats. Another similar study reported that melatonin significantly reduced the number of apoptotic cells in nucleus pulposus and epiphyseal cartilage of the spinal column and the expression of inducible nitric oxide synthase (iNOS), which increased after ovariectomy [52]. iNOS plays a pivotal role in the pathogenesis of osteoporosis. It generates nitric oxide, a free radical contributing to the imbalance between bone resorption and formation caused by estrogen depletion [52].

Recently, a randomized, double-blind, placebo-controlled clinical trial was carried out by Kotlarczyk *et al.* [14]. In this study, 18 perimenopausal women (ages 45–54) were randomized to receive melatonin (3 mg, per. os. *n* = 13) or placebo (*n* = 5) nightly for six months. The results showed no significant change in bone density, Type-I collagen cross-linked *N*-telopeptide (NTX), or osteocalcin (OC) between groups; however, the ratio of NTX:OC trended downward over time toward a ratio of 1:1 in the melatonin group, while the trend was not seen in the placebo group. NTX and OC are the bone turnover markers: NTX for bone resorption, and OC for bone formation. The ratio of NTX:OC trending downward to 1:1 in the melatonin group indicates that melatonin supplementation may restore imbalances in bone remodeling to prevent bone loss in perimenopausal women.

From these studies, we found that melatonin application markedly influenced the bone microenviroment and bone metabolism after ovariectomy or menopausal, suggesting its potential use in the prevention/treatment of osteoporosis.

## 4. Melatonin and Tooth

### 4.1. Possible Involvement of Melatonin in Tooth Development

Melatonin concentrations change in a specific manner during the lifespan of human [53]. Melatonin is a lipophilic hormone that crosses the placenta barrier easily, thus, prenatally, the fetal obtain melatonin from mothers [54]. During the first two weeks of life, melatonin could be detected in infant blood, but there was no daily rhythm [55,56]. The nocturnal rise of melatonin concentrations appears in the sixth to eighth week of life [53,55,57], and its circadian rhythm seems to be well established around three months of age [57]. After this period, the melatonin concentration continues to increase. The amplitude of the nocturnal peak in melatonin secretion reaches the highest levels in the ages between four and seven years [53]. Until the onset of puberty (about 10–12 years old), the melatonin concentrations begin to decline [1]. Interestingly, over this time period, teeth undergo a series of pivotal activities including histogenesis, development, eruption, replacement and maturity. The development of deciduous teeth and permanent teeth start from the second and fourth month of gestation, respectively. Deciduous teeth begin eruption in the sixt month of life, until 2.5–3 years of age all of deciduous teeth have erupted. From about six years old, permanent teeth begin to replace the deciduous teeth until 10–12 years old. Since the time course of melatonin secretion and the progression of tooth development run in parallel, the possible role of melatonin in tooth development should be worthy of study.

The most striking feature of the melatonin is the circadian rhythm which is controlled by the endogenous circadian clock, suprachiasmatic nucleus (SCN) and environmental light. Many studies also reported that tooth development exhibits circadian rhythmicity [58,59]. Periodic growth incremental lines are found universally in the dental tissues of animals, especially in the dentine and enamel, which reflect circadian rhythms of tooth growth [59,60]. A previous study had also demonstrated that SCN plays an important role in generating circadian dentine increments [59]. Complete lesion of the SCN led to a failure of the dentine increments’ appearance, and the authors presumed that this was associated with changes in hormones under tight circadian control [59]. Therefore, we presumed that melatonin may be involved in the development of circadian dental formation.

Recently, some experimental studies focused on the possible role of melatonin in tooth development have been performed. Kumasaka *et al.* [61] revealed that melatonin 1a receptor is expressed in secretory ameloblasts, the stratum intermedium and stellate reticulum cells, external dental epithelial cells, odontoblasts, and dental sac cells in the tooth germs of the mandibular third molar of human. An *in vitro* study showed that HAT-7, a rat dental epithelial cell line, expressed Mel1aR and its expression levels increased after the cells reached confluence. Moreover, our previous study [17] demonstrated that melatonin (physiological dose range) induced a dose-dependent reduction in rat dental papilla cell (rDPCs) proliferation, increased ALP activity, DSP expression, and mineralized matrix formation *in vitro. In vivo* melatonin inhibited dentine formation in rats. We also found that melatonin suppressed, in a basal medium, the activities of complex I and IV of mitochondrial respiratory chain, but enhanced these activities in an osteogenic medium. Our data strongly suggest a physiological role of melatonin in tooth development by regulating cellular processes in odontogenic cells, which may involve the modulation of mitochondria function.

### 4.2. Melatonin and Osseointegration of Dental Implant

Dentition defect and dentition missing are the most common dental diseases, especially in middle-aged and elderly people. There are various conventional restorative options for edentulous patients, such as removable dentures, fixed dentures, overdentures, *etc.* Unfortunately conventional denture wearers experience a number of problems on a daily basis, such as instability of their removable dentures, inability to comminute foods, decreased self-confidence, and so on [62]. Nowadays, implant-supported denture, a new technology for restoration, has addressed these daily problems. Thus, in the past several decades, implant-supported prosthesis has expanded to become a widely accepted treatment for the restoration of fully and partially edentulous patients [62].

The premise of the success of dental implant is osseointegration, which refers to the direct contact histologically between living bone and the surfaces of commercially pure titanium implants. It is critical for providing rigid fixation of a dental implant within the alveolar bone and promoting the long-term success of dental implants [63,64]. The most widely investigated topics include enhancing the success rate of the dental implant, minimizing the time of osseointegration, modifying implant surfaces, and seeking novel biomaterials to positively modulate the host/implant tissue response [65,66]. Several experimental investigations show that melatonin may be a potent biomimetic agent in the placement of endo-osseous dental implants. Cutando *et al.* [5] revealed that after a two-week treatment period, melatonin significantly increased the perimeter of bone that was in direct contact with the treated implants, bone density, new bone formation and inter-thread bone in comparison with control implants. During this study, they observed the increase in osteoblast proliferation brought about by melatonin in the peri-implant zone. The same outcome also was demonstrated by another study; Guardia *et al.* [67] found that after five- and eight-week treatment periods, melatonin significantly increased the inter-thread bone and new bone formation in comparison to control implants in both weeks. Moreover, in a further study performed by Calvo-Guirado *et al.* [68], the result showed that melatonin plus porcine bone significantly increased the perimeter of bone that was in direct contact with the treated implants, and that bone density and new bone formation increased in comparison with porcine bone alone around the implants. These actions of melatonin on osseointegration are of interest as it may be possible to apply melatonin to dental implant surgery as a biomimetic agent.

## 5. Conclusions

Melatonin, as an endogenous hormone, participates in many physiological and pharmacological processes. The above analyzed data indicate that melatonin may be involved in the development of the hard tissues bone and teeth. Decreased melatonin levels may be related to bone disease and abnormality. Due to its ability of regulating bone metabolism, enhancing bone formation, promoting osseointegration of dental plant and cell and tissue protection, melatonin may used as a novel mode of therapy for augmenting bone mass in bone diseases characterized by low bone mass and increased fragility, bone defect/fracture repair and dental implant surgery. The investigation of melatonin on tooth still insufficient and requires further research.
